# Supplementary material for: Fasting mimicking diet in mice delays cancer growth and reduces immunotherapy-associated cardiovascular and systemic side effects
Source: Nat Commun. 2023 Sep 8;14:5529. doi: 10.1038/s41467-023-41066-3 (PMC10491752; doi:10.1038/s41467-023-41066-3)
Supplement: Supplementary file 1 — Supplementary Information [file 41467_2023_41066_MOESM1_ESM.pdf]

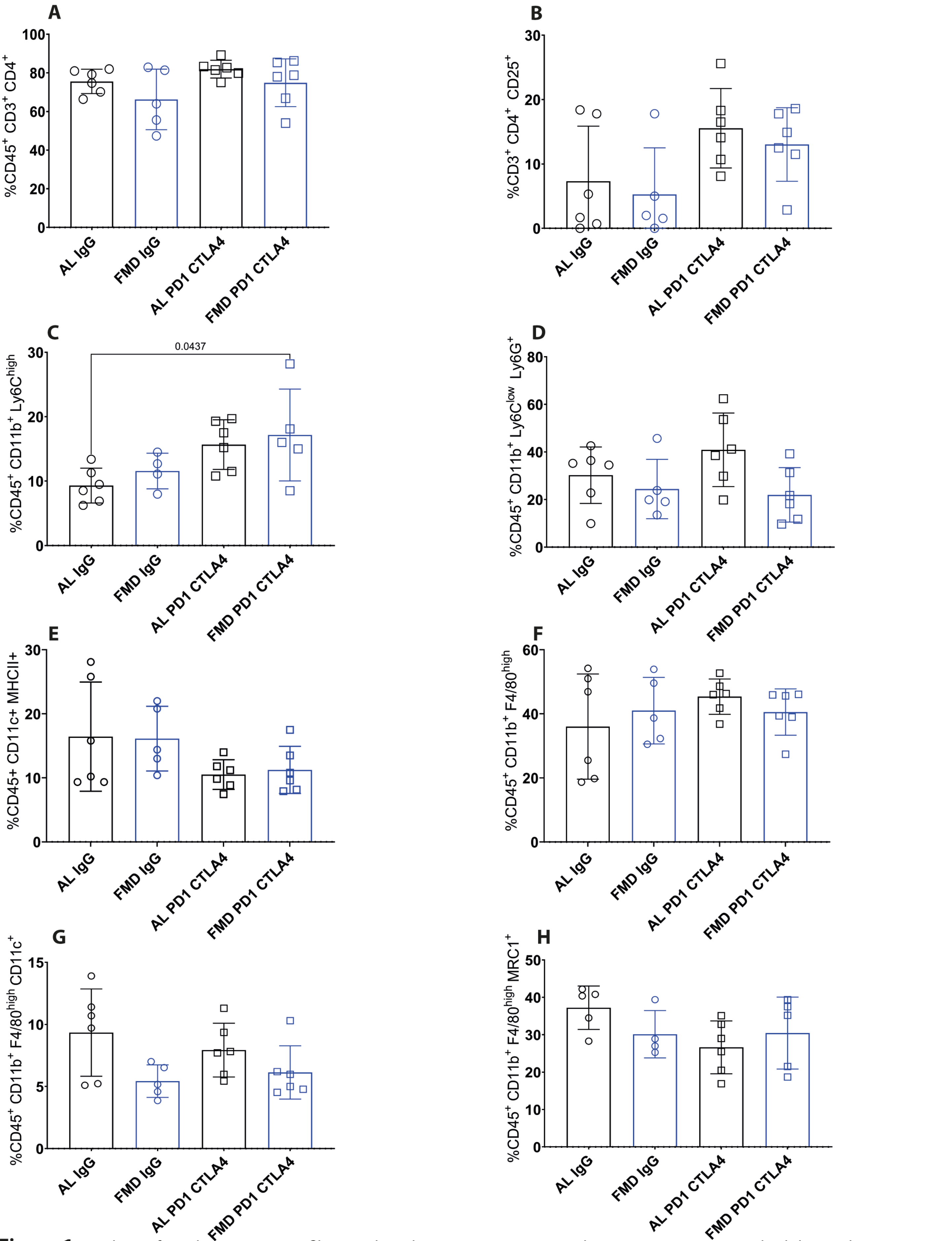

Suppl Figure 1. Analysis of B16 breast tumor infiltrating lymphocytes upon immunotherpy treatment in standard diet and FMD group. (**A**) CD45$^+$CD3$^+$CD4 $^+$T cell (AL NT n=6; FMD NT n=5; AL PD1 CTLA4 n=6; FMD PD1 CTLA4 n=6); (**B**) CD3$^+$CD4$^+$CD25 $^+$Treg cells (AL NT n=6; FMD NT n=5; AL PD1 CTLA4 n=6; FMD PD1 CTLA4 n=6); (**C**) CD45$^+$CD11b$^+$Ly6C^{high} M-MDSC (AL NT n=6; FMD NT n=4; AL PD1 CTLA4 n=6; FMD PD1 CTLA4 n=5); **(D)** CD45$^+$CD11b$^+$Ly6C^{low} Ly6G^{high}PMN-MDSC (AL NT n=6; FMD NT n=5; AL PD1 CTLA4 n=6; FMD PD1 CTLA4 n=6); (**E**) CD45$^+$ CD11c$^+$MHCII$^+$ dendritic cell (AL NT n=6; FMD NT n=5; AL PD1 CTLA4 n=6; FMD PD1 CTLA4 n=6); (**F**)CD45$^+$ CD11b$^+$F4/80^{high} macrophage (AL NT n=6; FMD NT n=5; AL PD1 CTLA4 n=6; FMD PD1 CTLA4 n=6), (**G**) CD45$^+$CD11b$^+$F480^{high}CD11c$^+$ M1 (AL NT n=6; FMD NT n=5; AL PD1 CTLA4 n=6; FMD PD1 CTLA4 n=6); (**H**) CD45$^+$CD11b$^+$F480^{high}MRC1$^+$ M2 macrophage (AL NT n=5; FMD NT n=4; AL PD1 CTLA4 n=6; FMD PD1 CTLA4 n=5). Statistical analysis was performed using one-way analysis of variance (ANOVA).). P values were determined by One-way ANOVA with Tukey's post analysis.Differences were considered significant when $P \leq 0.05$. All data are represented as mean ± SEM. Source data are provided as a Source Data file.

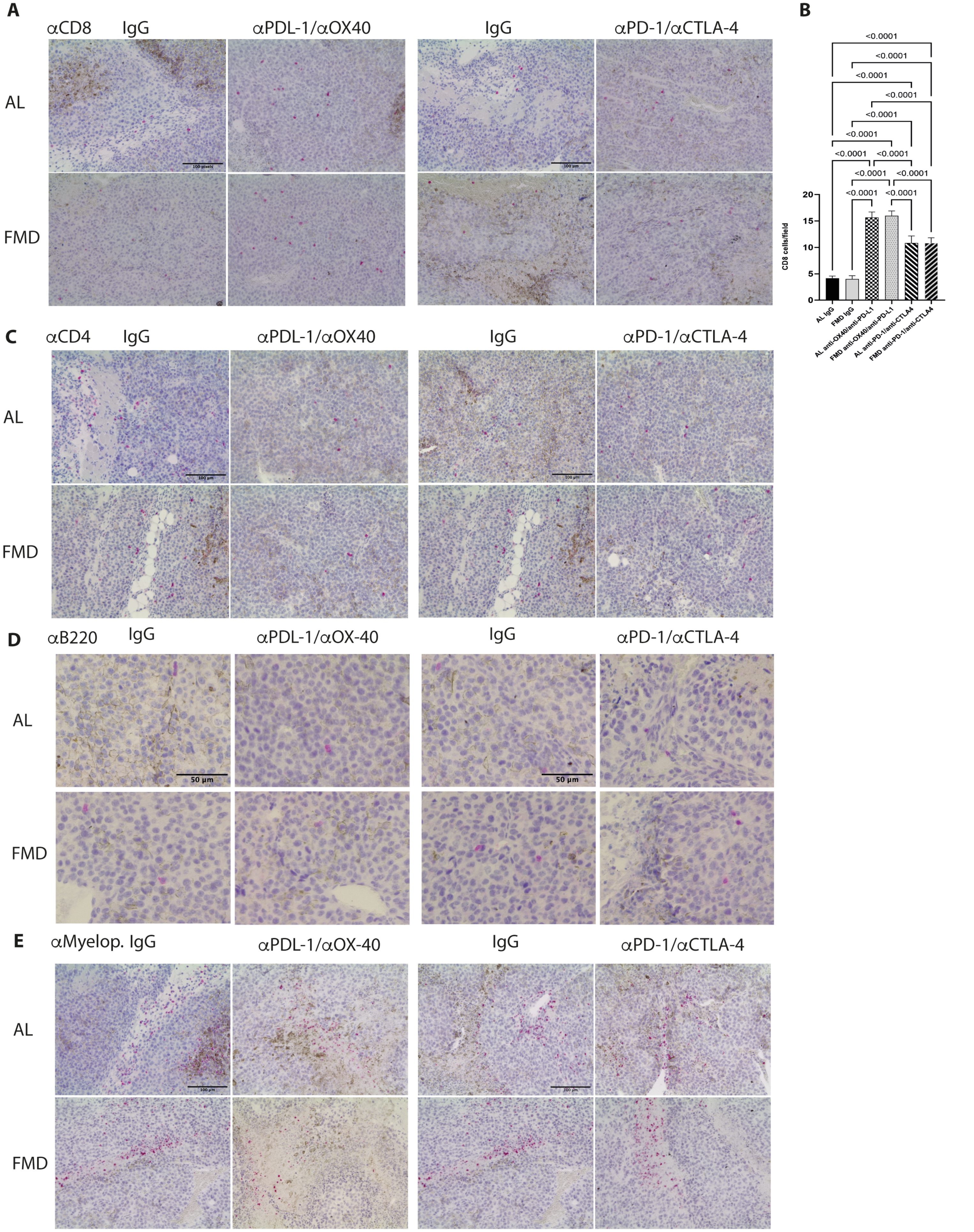

Suppplementary Figure 2. IHC analysis of immuneinfiltrate in B16 melanoma tumor section. Representative images of tumor section stained with: A) CD8, C) CD4, D) B220, E) Myeloperoxidase. The images were obtained under 20× magnification. The scale bar was 100 µm. B) Quantification of CD8 T cells infiltration in tumor sections. Statistical analysis was performed using one-way analysis of variance (ANOVA) (n = 3 tumors; 7 random fields/tumor). P values were determined by One-way ANOVA with Tukey's post analysis. Differences were considered significant when P≤0.05. All data are represented as mean ± SEM.

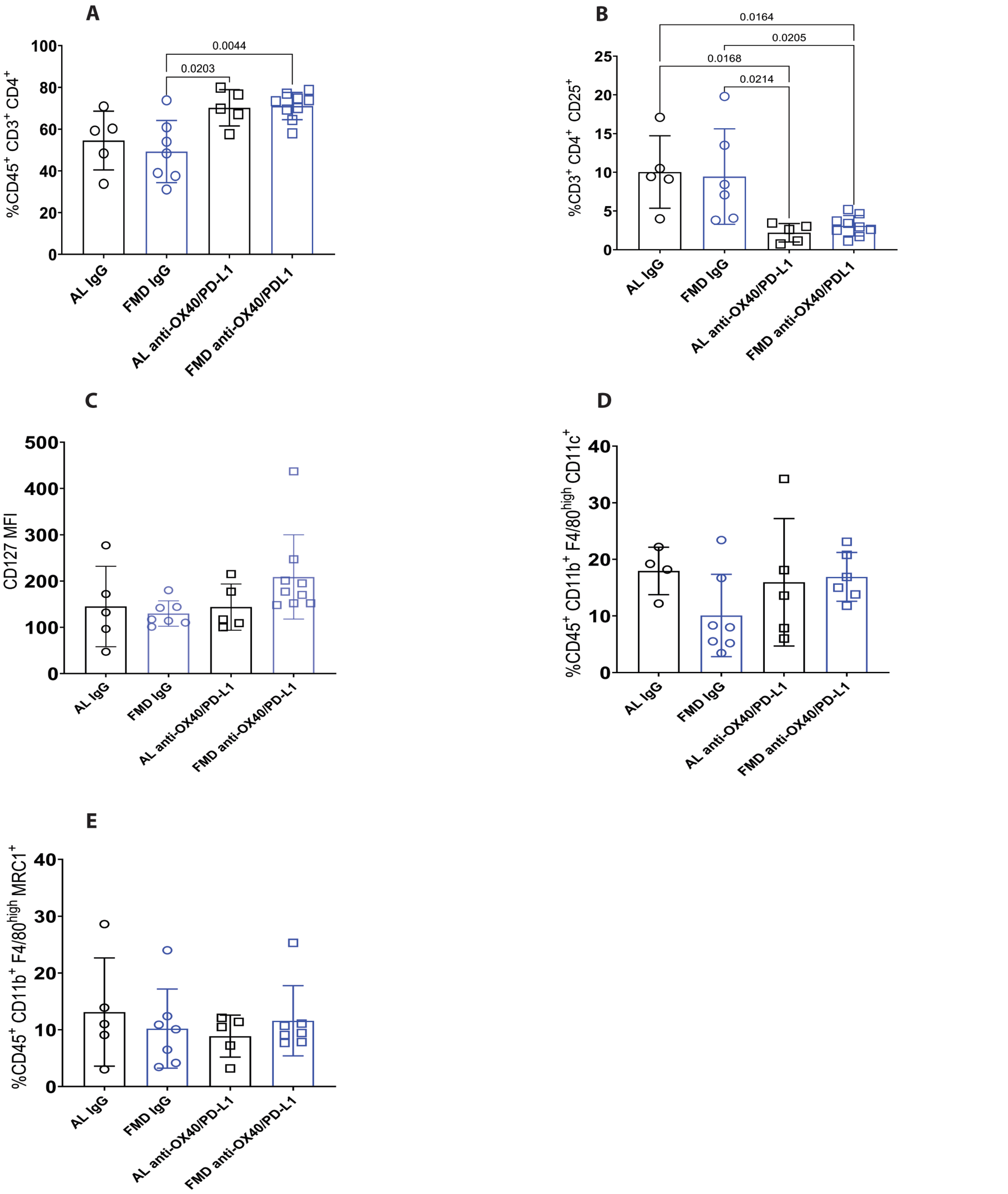

Suppl Figure 3. Analysis of B16 breast tumor infiltrating lymphocytes upon immunotherpy treatment in standard diet and FMD group. (**A**) $CD45^+CD3^+CD4^+$ T cell (AL NT n=5; FMD NT n=7; AL anti-OX40/PD-L1 n=5; FMD anti-OX40/PD-L1 n=9); (**B**) $CD3^+CD4^+CD25^+$ Treg cells (AL NT n=5; FMD NT n=6; AL anti-OX40/PD-L1 n=5; FMD anti-OX40/PD-L1 n=9); (**C**) mean fluorescence intensity (MFI) of CD127 in $CD3^+CD8^+CD44^+$ effector T cells (AL NT n=5; FMD NT n=7; AL anti-OX40/PD-L1 n=5; FMD anti-OX40/PD-L1 n=9); (**D**) $CD45^+CD11b^+F480^{high}CD11c^+$ (AL NT n=4; FMD NT n=7; AL anti-OX40/PD-L1 n=5; FMD anti-OX40/PD-L1 n=6), (**E**) $CD45^+CD11b^+F480^{high}MRC1^+$ (AL NT n=5; FMD NT n=7; AL anti-OX40/PD-L1 n=5; FMD anti-OX40/PD-L1 n=7). Statistical analysis was performed using one-way analysis of variance (ANOVA). P values were determined by One-way ANOVA with Tukey's post analysis.Differences were considered significant when $P \leq 0.05$. All data are represented as mean ± SEM.

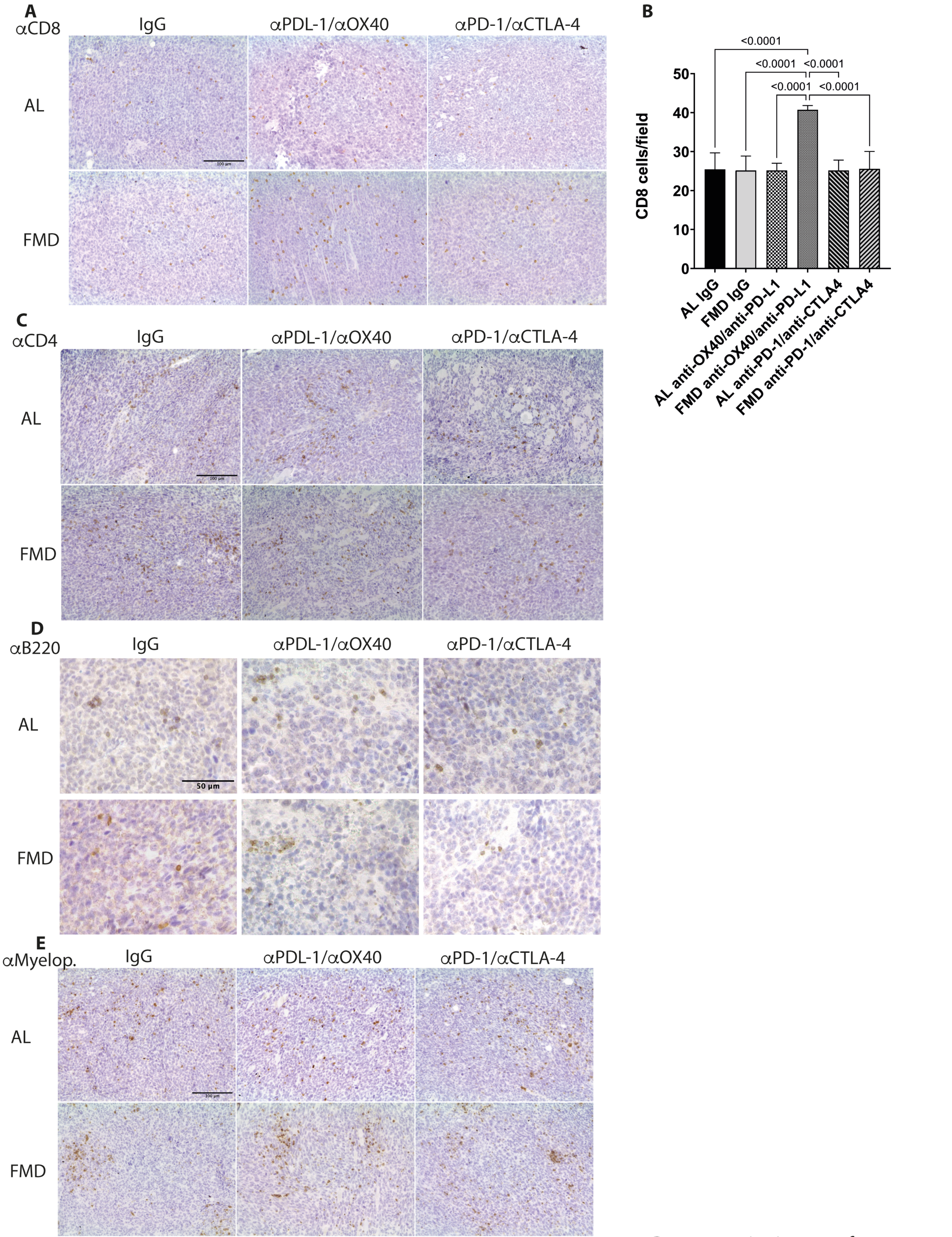

Suppplementary Figure 4. IHC analysis of immuneinfiltrate in LLC1 lung tumor section. Representative images of tumor section stained with: A) CD8, C) CD4, D) B220, E) Myeloperoxidase (n=5). The images were obtained under 20× magnification. The scale bar was 100 μm . B) Quantification of CD8 T cells infiltration in tumor sections (n = 3 tumors; 7 random fields/ tumor). Statistical analysis was performed using one-way analysis of variance (ANOVA).). P values were determined by One-way ANOVA with Tukey's post analysis. Differences were considered significant whenP≤0.05. All data are represented as mean ± SEM.

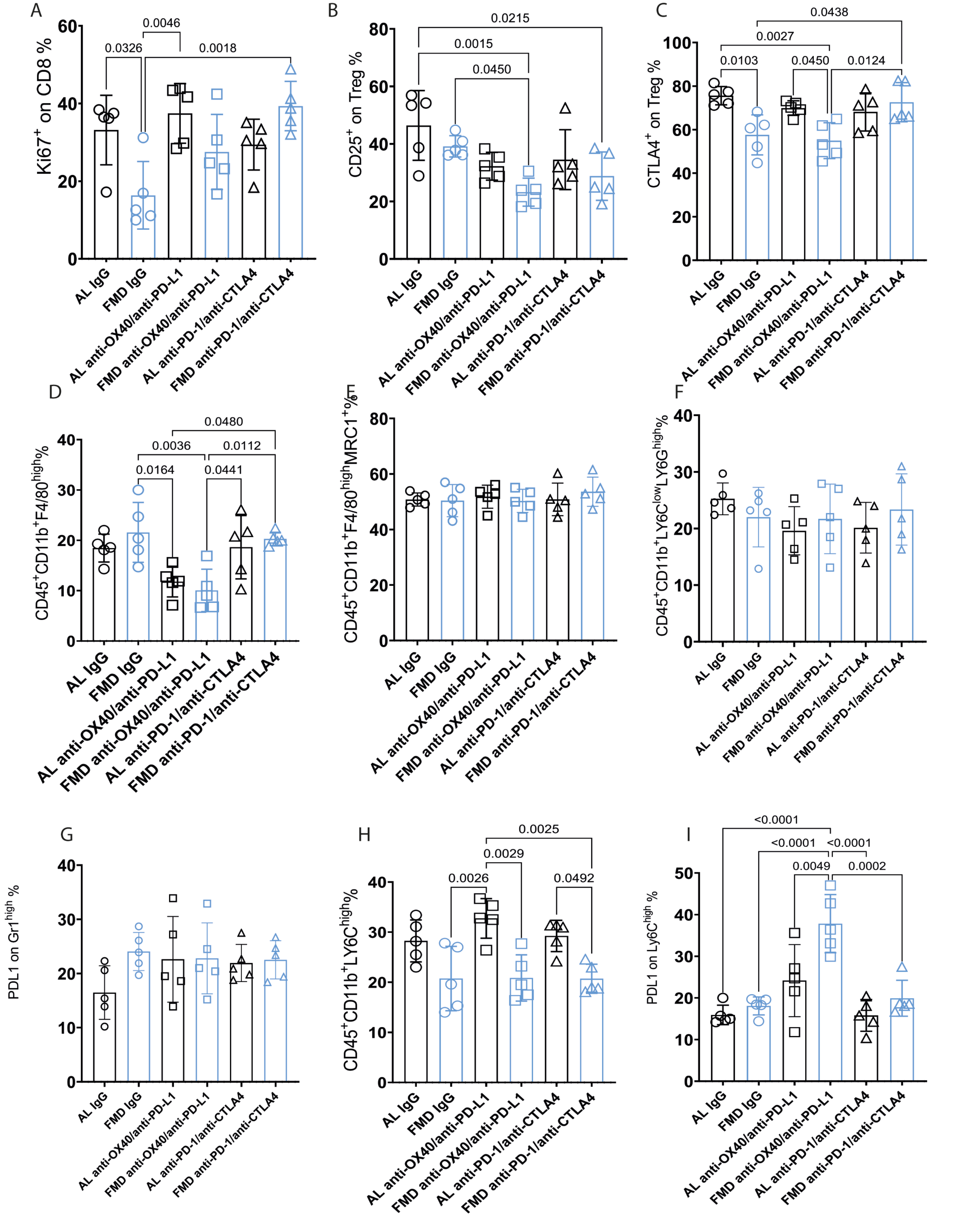

Supplementary Figure 5. Analysis of LLC1 lung tumor infiltrating lymphocytes upon immunotherpy treatment in standard diet and FMD group. A) $KI67^+$ on $CD8^+$ T cells (n=5); B) $CD25^+$ on Treg cells (n=5); C) CTLA-4^+ on Treg cells; D) $CD45^+$ $CD11b^+F4/80^{high}$ macrophage (n=5); E) $CD206^+$ on $CD45^+$ $CD11b^+F4/80^{high}$ macrophage (n=5); F) $CD45^+CD11b^+GR1^{high}$ PMN-MDSC (n=5) G) PD-$L1^+$ on $GR1^{high}$ PMN-MDSC (n=5); H) $CD45^+CD11b^+Ly6C^{high}$ M-MDSC (n=5); I) PD-$L1^+$ on $Ly6C^{high}$ M-MDSC (n=5). Statistical analysis was performed using one-way analysis of variance (ANOVA). P values were determined by One-way ANOVA with Tukey's post analysis.Differences were considered significant when $P \leq 0.05$. All data are represented as mean ± SEM.

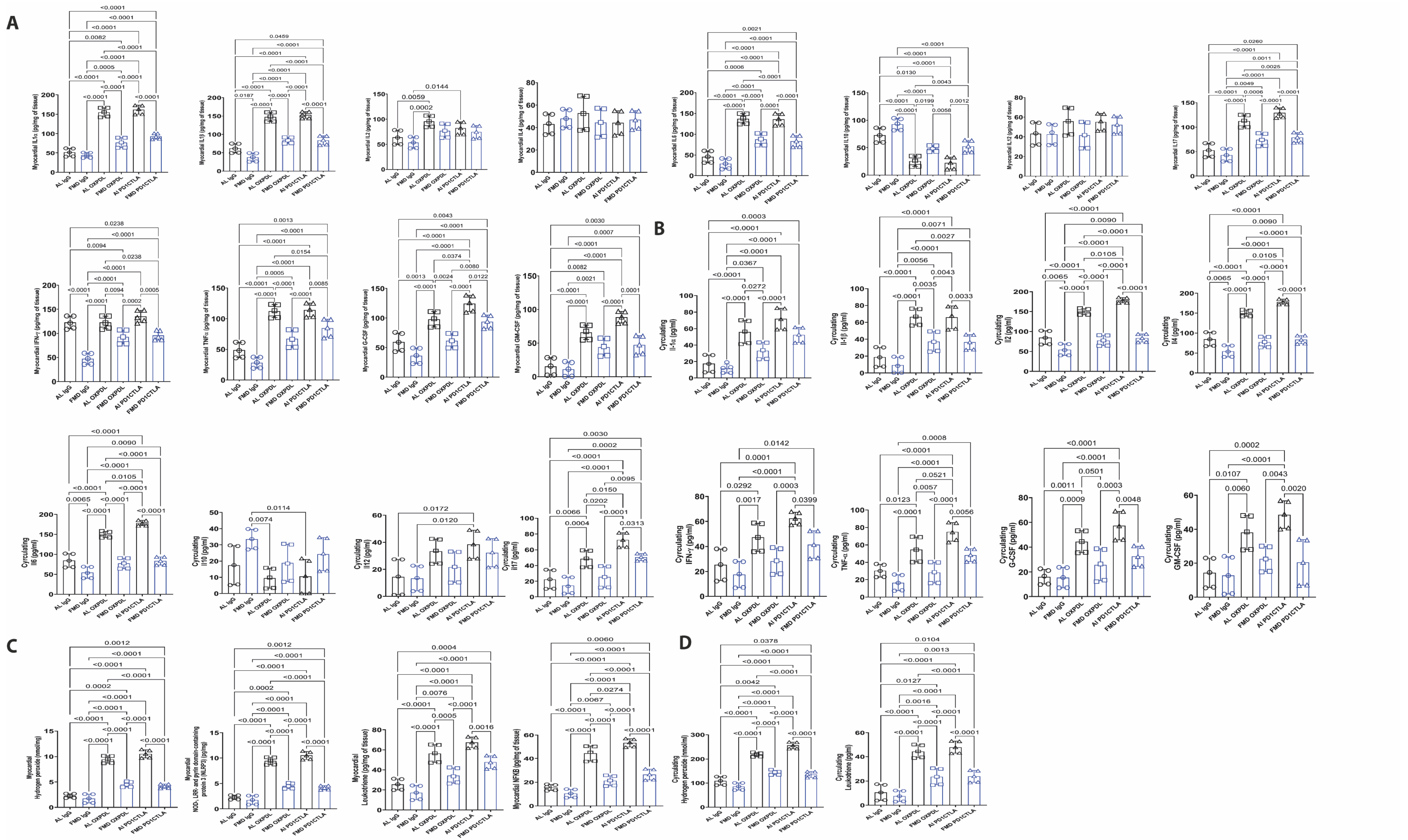

Supplementary Figure 6. FMD affects pro- and anti-inflammatory cytokine secretion upon immunotherapy treatment and attenuate Reactive Oxygen Species (ROS), NLRP-3 inflammasome, leukotrienes and NF-kB expression in heart and plasma. Cytokines level (pg/ml) in heart (**A**) and plasma (**B**). ROS, NLRP-3 inflammasome, leukotrienes and NF-kB expression in heart (**C**) and plasma (**D**). IL-1α,1β, 2, 4, 6, 12, 17α: interleukin -1α,1β, 2, 4, 6, 12, 17α; IFN-γ: interferon γ; TNF-α: tumor necrosis factor a; G-CSF: granulocyte colony stimulating factor; GM-CSF: granulocyte-macrophage colony stimulating factor (n=5). Statistical analysis was performed using one-way analysis of variance (ANOVA). P values were determined by One-way ANOVA with Tukey's post analysis.Differences were considered significant when $P<0.05$. All data are represented as mean ± SEM. 'Source data are provided as a Source Data file

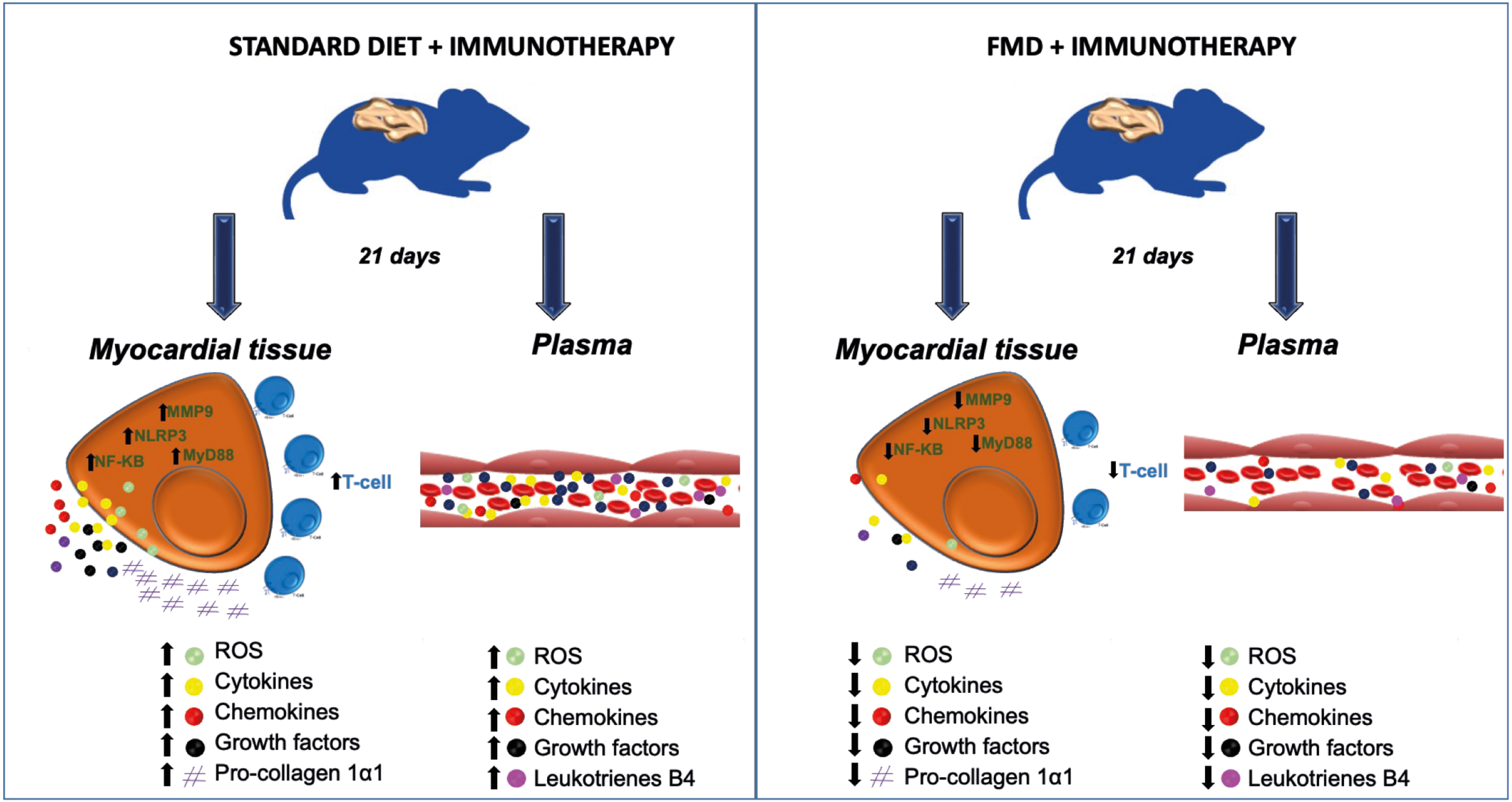

Supplementary Figure 7. Overall picture of the study demonstrating FMD decreases immune $CD3^+$ and $CD8^+$ lymphocytes and NLRP-3, Pro-collagen 1α1, MMP-9 and NF-KB expression in myocardial tissue of C57BL/6J melanoma bearing mice treated for 21 days with anti-OX40/anti PD-L1 and anti-PD-1/anti-CTLA-4 therapy. Systemic and myocardial chemokines, cytokines and growth factors(IL-1α, IL-1β, IL-6, IL17-α, G-CSF, and GM-CSF levels were also reduced in FMD group vs AI group.

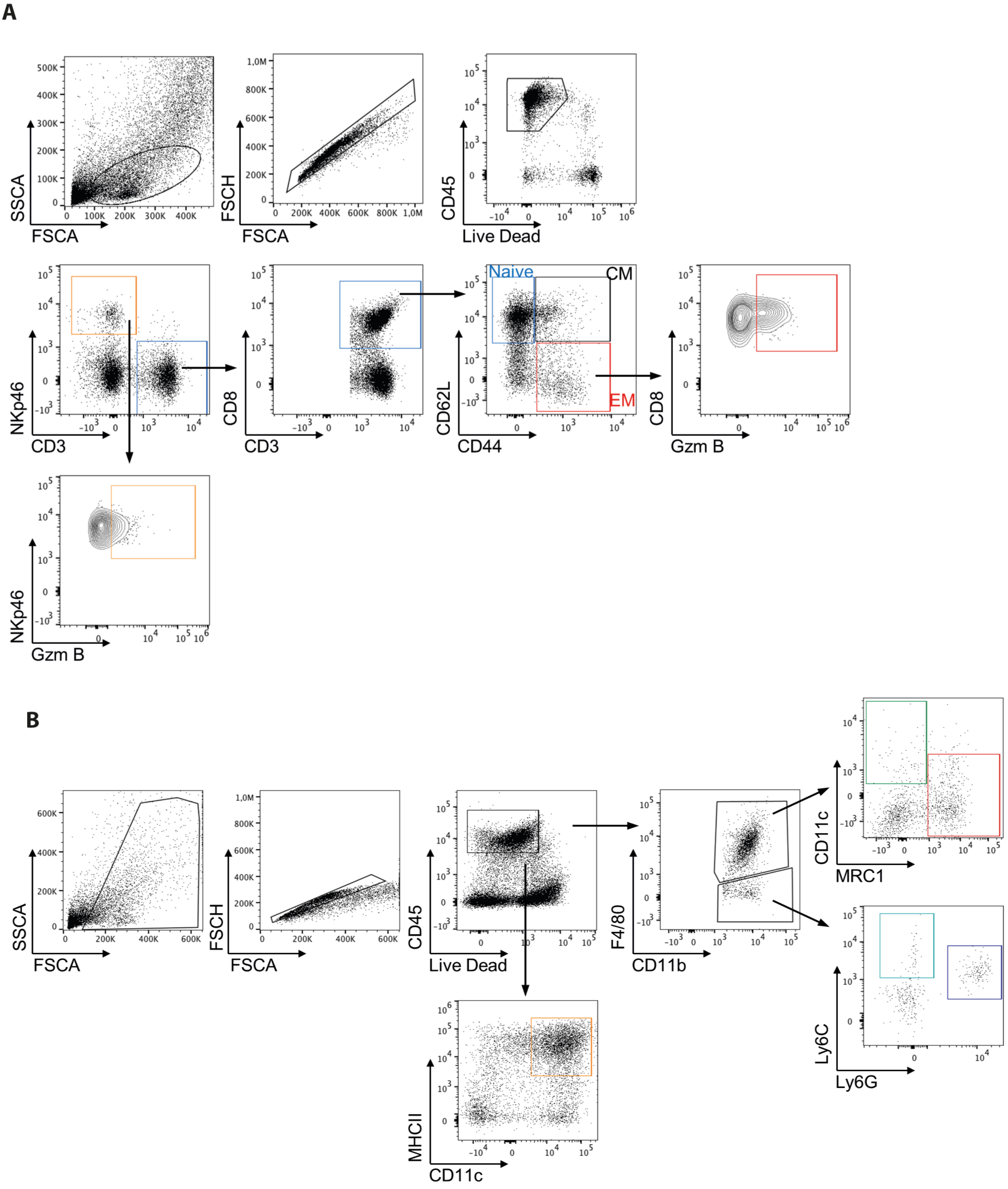

Supplementary Figure 8. Gating strategy for FACS analysis of immune infiltrate. **A**) Gating strategy for CD8 lymphocyets and NK cells(Figure 1 D-F and Figure 2 D-F). Among viable CD45$^+$ cells, NK cells were gated as NKp46$^+$ and T cells were gated as CD3$^+$. Among T cells, CD8$^+$ effector memory T cells were gated as CD44$^+$ CD62L$^-$.**B**) Gating strategy for myeloid cells (Figure 3 A-D). Among CD45$^+$ cells, dendritic cells (DC) were gated as CD11c$^+$ MHCII$^+$ and macrophages were gated as F4/80$^+$ CD11b$^+$. PMN-MDSC and M-MDSC cells were identified among F4/80– CD11b$^+$ cells according to the expression of Ly6C and Ly6G.
