# Supplementary material for: Fasting mimicking diet in mice delays cancer growth and reduces immunotherapy-associated cardiovascular and systemic side effects
Source: Nat Commun. 2023 Sep 8;14:5529. doi: 10.1038/s41467-023-41066-3 (PMC10491752; doi:10.1038/s41467-023-41066-3)
Supplement: Supplementary file 3 — Reporting Summary [file 41467_2023_41066_MOESM3_ESM.pdf]

nature portfolio

Corresponding author(s): Valter D Longo

Last updated by author(s): 2023/09/06

Reporting Summary

Nature Portfolio wishes to improve the reproducibility of the work that we publish. This form provides structure for consistency and transparency in reporting. For further information on Nature Portfolio policies, see our Editorial Policies and the Editorial Policy Checklist.

Statistics

For all statistical analyses, confirm that the following items are present in the figure legend, table legend, main text, or Methods section.

n/a	Confirmed	
☐	☒	The exact sample size (*n*) for each experimental group/condition, given as a discrete number and unit of measurement
☐	☒	A statement on whether measurements were taken from distinct samples or whether the same sample was measured repeatedly
☐	☒	The statistical test(s) used AND whether they are one- or two-sided<br>*Only common tests should be described solely by name; describe more complex techniques in the Methods section.*
☒	☐	A description of all covariates tested
☐	☒	A description of any assumptions or corrections, such as tests of normality and adjustment for multiple comparisons
☐	☒	A full description of the statistical parameters including central tendency (e.g. means) or other basic estimates (e.g. regression coefficient) AND variation (e.g. standard deviation) or associated estimates of uncertainty (e.g. confidence intervals)
☐	☒	For null hypothesis testing, the test statistic (e.g. *F*, *t*, *r*) with confidence intervals, effect sizes, degrees of freedom and *P* value noted<br>*Give P values as exact values whenever suitable.*
☒	☐	For Bayesian analysis, information on the choice of priors and Markov chain Monte Carlo settings
☒	☐	For hierarchical and complex designs, identification of the appropriate level for tests and full reporting of outcomes
☐	☒	Estimates of effect sizes (e.g. Cohen's *d*, Pearson's *r*), indicating how they were calculated

Our web collection on statistics for biologists contains articles on many of the points above.

Software and code

Policy information about availability of computer code

Data collection	-FACS analysis: samples were acquired by Attune NxT flow cytometer;<br>-Colorimetric and Fluorimetric assay(absorbance): Tecan Infinite M200 plate-reader
Data analysis	-FACS data were analyzed by FlowJo software v10 (Treestar)<br>-Statisitcal analysis and graphing were done using GraphPad Prism(9.0)<br>-Sample size was determined using GPower 3.1 software (Power and Sample size calculation-Vanderbilt University)

For manuscripts utilizing custom algorithms or software that are central to the research but not yet described in published literature, software must be made available to editors and reviewers. We strongly encourage code deposition in a community repository (e.g. GitHub). See the Nature Portfolio guidelines for submitting code & software for further information.

Data

Policy information about availability of data

All manuscripts must include a data availability statement. This statement should provide the following information, where applicable:

- Accession codes, unique identifiers, or web links for publicly available datasets
- A description of any restrictions on data availability
- For clinical datasets or third party data, please ensure that the statement adheres to our policy

All data generated or analysed during this study has been deposited in Figshare, ???

Research involving human participants, their data, or biological material

Policy information about studies with human participants or human data. See also policy information about sex, gender (identity/presentation), and sexual orientation and race, ethnicity and racism.

Reporting on sex and gender	N/A
Reporting on race, ethnicity, or other socially relevant groupings	N/A
Population characteristics	N/A
Recruitment	N/A
Ethics oversight	N/A

Note that full information on the approval of the study protocol must also be provided in the manuscript.

Field-specific reporting

Please select the one below that is the best fit for your research. If you are not sure, read the appropriate sections before making your selection.

[x] Life sciences [ ] Behavioural & social sciences [ ] Ecological, evolutionary & environmental sciences

For a reference copy of the document with all sections, see nature.com/documents/nr-reporting-summary-flat.pdf

Life sciences study design

All studies must disclose on these points even when the disclosure is negative.

Sample size	For in vivo experimens, sample size estimation was performed with G.Power software using ANOVA repeated measurement, within-between interaction.A power analysis with significance level a = 0.05, (assuming a large effect size f = 0.4) indicated that n=8 mice per group are needed to achieve a power (1 – b) of 0.9 which is considered adequate to detect difference between means at least of two groups. Therefore for melanoma and lung tumors xenograft study, each group includes at least 8 mice. No statistical methods were used to predetermine sample sizes for FACS analyzes or for the quantification of cytokines and lymphocytic infiltrates, but our sample sizes were determinated based on our previous experience and are similar to those reported in previous publications.
Data exclusions	No data were excluded for pre-established criteria
Replication	We standardized the protocols for all in vivo experiments using the same time frame, drug concentration and cell number. Some in vivo experiments were repeated and confirmed by other operators or have generated the same results obtained by other operators and already published. All experiments with multiple biological replicates are indicated in the figure legends.All in vitro experiments were replicated three times, obtaining successful replication of the results
Randomization	Melanoma and lung cancer xenograft Mice were randomly assigned to different groups at beginning of each experiment. However, covariates including sex and age of animals were identical in groups.
Blinding	For in vivo studies, blinding during animal experiments was not possible because mice underwent a specific diet supply and daily treatment. Operators were unblinded since they performed dietary and pharmacological treatment and treatment group are clearly indicated for each animal box.

Reporting for specific materials, systems and methods

We require information from authors about some types of materials, experimental systems and methods used in many studies. Here, indicate whether each material, system or method listed is relevant to your study. If you are not sure if a list item applies to your research, read the appropriate section before selecting a response.

Materials & experimental systems

n/a	Involved in the study
☐	☒ Antibodies
☐	☒ Eukaryotic cell lines
☒	☐ Palaeontology and archaeology
☐	☒ Animals and other organisms
☒	☐ Clinical data
☒	☐ Dual use research of concern
☒	☐ Plants

Methods

n/a	Involved in the study
☒	☐ ChIP-seq
☐	☒ Flow cytometry
☒	☐ MRI-based neuroimaging

Antibodies

Antibodies used

Anti-mouse/human CD44, PB (IM7) Biolegend cat# 103020 diluted 1:200
Anti- mouse FoxP3, eFluor 506 (FJK-16s) eBioscience cat# 69-5773-82 diluted 1:100
Anti-mouse CD274 (PD-L1, B7-H1), Biotin (1-111A) eBioscience cat# 13-9971-81 diluted 1:400
Anti-mouse Eomes, PE (Dan11mag) eBioscience cat# 12-4875-82 diluted 1:50
Anti-mouse CD127 (IL-7Rα), PE (A7R34) Biolegend cat# 135010 diluted 1:200
Anti-mouse CD8a, PerCP-Vio700 (53-6.7) Miltenyi Biotec cat# 130-120-756 diluted 1:50
anti-mouse CD185 (CXCR5), PE/Cy7 (L138D7) Biolegend cat# 145516 diluted 1:400
Anti-mouse CD335 (NKp46), PE-eFluor 610 (29A1.4) eBioscience cat# 61-3351-82 diluted 1:200
Anti-mouse CD366 (Tim-3), PE/Dazzle 594 (B8.2C12) Biolegend cat# 134013 diluted 1:400
Anti-mouse T-bet, AF 647 (4B10) Biolegend cat# 644804 diluted 1:500
Anti-mouse/human KLRG1, APC (2F1/KLRG1) Biolegend cat# 138412 diluted 1:400
Anti-mouse Ly108, APC (330-AJ) Biolegend cat# 134610 diluted 1:400
Anti-mouse CD3, AF 700 (17A2) eBioscience cat# 56-0032-82 diluted 1:500
Anti-mouse MRC1, AF 700 (MR6F3) eBioscience cat# 56-2061-82 diluted 1:400
Anti-mouse CD25, APC/Cy7 (3C7) Biolegend cat# 101918 diluted 1:200
Anti-mouse PD1, Biotin (RMP1-30) Biolegend cat# 109106 diluted 1:500
Anti-mouse CD11c, APC-Vio770 (N418) Miltenyi Biotec cat# 130-107-461 diluted 1:50
Anti- mouse TCF1/TCF7, AF 488 (C63D9) Cell Signaling Technology cat# BK6444S diluted 1:50
Anti-mouse CD11b, VioBlue (REA592) Miltenyi Biotec cat# 130-113-810 diluted 1:50
Anti-mouse CD4, VioBright FITC (REA604) Miltenyi Biotec cat# 130-118-692 diluted 1:50
Anti-mouse Granzyme B, FITC (REA226) Miltenyi Biotec cat# 130-118-341 diluted 1:50
Anti-mouse Ly-6C, FITC (REA796) Miltenyi Biotec cat# 130-111-915 diluted 1:50
Anti-mouse MHC Class II, PE (REA813) Miltenyi Biotec cat# 130-112-231 diluted 1:50
Anti-mouse CD62L, PE (MEL-14) Biolegend cat# 104407 diluted 1:500
Anti-mouse CTLA-4, BV421 (UC10-4B9) Biolegend cat# 106311 diluted 1:40
Anti-mouse Ly-6G, PerCP-Vio700 (REA526) Miltenyi Biotec cat# 130-117-500 diluted 1:50
Anti-mouse CD45, PE-Vio770 (REA737) Miltenyi Biotec cat# 130-110-661 diluted 1:50
Anti-mouse F4/80, APC (REA126) Miltenyi Biotec cat# 130-116-525 diluted 1:50
Antibodies in vivo
anti-mouse OX40 (CD134) BioXcell cat# BE0031 100ug/mouse
anti-mouse PD-L1 (B7-H1) BioXcell cat# BE0101 100ug/mouse
anti-mouse CTLA4 (CD152) BioXcell cat# BP0032 100ug/mouse
anti-mouse PD-1 (RMP1-14) BioXcell cat# BE0146 100ug/mouse
Antibodies IHC
Anti-CD3 Abcam cat# Ab16669 diluted 1:100
Anti- CD8α Abcam cat# Ab217344 diluted 1:100
Anti-Myeloperoxidase Abcam cat# Ab208670 diluted 1:100
Anti-B220 Bd bioscience cat# 553928 diluted 1:100

Validation

Most of the antibodies have been validated on gene ko cell lines or on cell lines overexpressing the gene of interest by us or by the manufacturer, as reported in the technical data sheet.
FACS antybodies:
Anti-mouse/human CD44, PB (IM7): Clone IM7 has been reported to recognize an epitope common to alloantigens and all isoforms of CD44(17,18) that is located between amino acids 145 and 18620. This clone has been quality tested for FC and verified for immunocytochemistry (ICC) and frozen immunohistochemistry (IHC-F). Lee JW, et al. 2006. Nature Immunol. 8:181; Wang XY, et al. 2008. Blood 111:2436.

Anti- mouse FoxP3, eFluor 506 (FJK-16s)
Applications Reported: This FJK-16s antibody has been reported for use in intracellular staining followed by flow cytometric analysis.
Applications Tested: This FJK-16s antibody has been tested by intracellular staining and flow cytometric analysis of mouse splenocytes using the Foxp3/Transcription Factor Staining Buffer Set (cat. 00-5523) and protocol. Shi LZ et al.Interdependent IL-7 and IFN-γ signalling in T-cell controls tumour eradication by combined α-CTLA-4+α-PD-1 therapy. Nat Commun. 7:12335(2016)

CD274 (PD-L1, B7-H1) Monoclonal Antibody (1-111A)
Applications Reported: The 1-111A antibody has been reported for use in flow cytometric analysis. Applications Tested: The 1-111A antibody has been tested by flow cytometric analysis of mouse splenocytes. Huber S et al. Alternatively activated macrophages inhibit T-cell proliferation by Stat6-dependent expression of PD-L2. Blood 116(17):3311-20 (2010).

EOMES Monoclonal Antibody (Dan11mag)
Applications Reported: This Dan11mag antibody has been reported for use in intracellular staining followed by flow cytometric analysis. Applications Tested: This Dan11mag antibody has been tested by intracellular flow cytometric analysis of mouse splenocytes using the Foxp3/Transcription Factor Staining Buffer Set (cat. 00-5523) and protocol. Campesato LF et al. Blockade of the AHR restricts a Treg-macrophage suppressive axis induced by L-Kynurenine. Nat Commun. 11(1):4011 (2020).

PE anti-mouse CD127 (IL-7Rα) reacts with mouse CD127, the high affinity alpha subunit of the mouse IL-7 receptor, and has been tested by flow cytometric analysis of mouse thymocytes and splenocytes.This antibody is quality control tested by immunofluorescent staining with flow cytometric analysis. Garo LP, et al. 2021. Nat Commun. 12:2419

CD8a Antibody, anti-mouse, PerCP-Vio® 700 Clone 53-6.7 is specific for the mouse CD8a antigen, also known as Ly-2, which is expressed on cytotoxic T cells. It has been tested by flow cytometric analysis of mouse thymocytes and splenocytes. In order to compare the epitope specificity of an antibody, the clone being used is compared with other known clones recognizing the same antigen in a competition assay. Zhao Z et al. Francisella tularensis induces Th1 like MAIT cells conferring protection against systemic and local infection. Nat Commun. 12(1):4355 (2021)

PE/Cyanine7 anti-mouse CD185 (CXCR5) Antibody reacts with CD185, also known as CXCR5, which is is expressed on B cells and a subset of T cells in the spleen. It has been tested by flow cytometric analysis of mouse thymocytes and splenocytes. Cohen CA, et al. 2021. Nat Commun. 12:4678.

CD335 (NKp46) Monoclonal Antibody (29A1.4) recognizes mouse NKp46, also known as CD335. CD335, a member of the natural cytotoxicity receptor (NCR) family, is a glycoprotein with 2 Ig-like domains and a short cytoplasmic tail. Expression of CD335 is uniquely found on NK cells. Applications Reported: This 29A1.4 antibody has been reported for use in flow cytometric analysis. This 29A1.4 antibody has been tested by flow cytometric analysis of mouse splenocytes. Sobecki M et al. NK cells in hypoxic skin mediate a trade-off between wound healing and antibacterial defence. Nat Commun. 12(1):4700 (2021)

PE/Dazzle™ 594 anti-mouse CD366 (Tim-3) Antibody reacts with CD366 (Tim-3) is a transmembrane protein also known as T cell immunoglobulin and mucin domain containing protein-3, expressed at high levels on Th1 lymphocytes and CD11b+ macrophages. This antibody is quality control tested by immunofluorescent staining with flow cytometric analysis. This antibody has been tested by flow cytometric analysis of mouse splenocytes. Darragh LB, et al. 2022. Nat Commun. 13:7015.

Alexa Fluor® 647 anti-T-bet Antibody reacts with T-box transcription factor T-bet, a "master regulator" of Th1 lymphoid development, highly expressed in hematopoietic cells including stem cells, NK cells, B cells, and T cells. This antibody is quality control tested by intracellular immunofluorescent staining using our True-Nuclear™ Transcription Factor Staining Protocol. This antibody has been tested by flow cytometric analysis of mouse splenocytes. Vogel AB, et al. 2021. Nature. 592:283.

APC anti-mouse/human KLRG1 (MAFA) Antibody reacts with Killer cell lectin-like receptor G1 (KLRG1), a type II membrane glycoprotein, expressed on subsets of CD8+ and CD4+ cells, including CD4+ and CD8+ effector/memory cells, potent regulatory CD4+ T cells. This antibody is quality control tested by immunofluorescent staining with flow cytometric analysis. This antibody has been tested by flow cytometric analysis of mouse splenocytes. Kobayashi T, et al. 2019. Cell. 176:982.

APC anti-mouse Ly108 Antibody reacts with Ly108, also known as SLAMF6 and NTB-A (NK cell, T cell, B cell antigen), one of the members in The Signaling Lymphocytic Activation Molecule (SLAM) family of immune receptors, expressed on T cells, B cells, macrophages, dendritic cells, NK cells, and granulocytes. This antibody is quality control tested by immunofluorescent staining with flow cytometric analysis. Di Pilato M, et al. 2021. Cell. 184(17)

CD3 Monoclonal Antibody (17A2), Alexa Fluor™ 700, reacts with the mouse CD3 complex, expressed by thymocytes in a developmentally regulated manner and by all mature T cells. Applications Reported: This 17A2 antibody has been reported for use in flow cytometric analysis. Applications Tested: This 17A2 antibody has been tested by flow cytometric analysis of mouse thymocyte and splenocyte suspensions. Wiernicki B et al. Cancer cells dying from ferroptosis impede dendritic cell-mediated anti-tumor immunity. Nat Commun. 13(1):3676 (2022)

CD206 (MMR) Monoclonal Antibody (MR6F3), Alexa Fluor™ 700, recognizes mouse CD206 also known as Macrophage Mannose Receptor (MMR) or Mannose Receptor C, Type 1 (MRC1), type 1 integral membrane glycoprotein receptor that is present in macrophages, some dendritic cells, as well as liver and lymphoid endothelial cells. Applications Reported: This MR6F3 antibody has been reported for use in flow cytometric analysis, and intracellular staining followed by flow cytometric analysis. Applications Tested: This MR6F3 antibody has been tested by intracellular staining and flow cytometric analysis of mouse resident peritoneal exudate cells using the Intracellular Fixation and Permeabilization Buffer. Chen J et al. Meningeal lymphatics clear erythrocytes that arise from subarachnoid hemorrhage. Nat Commun. 11(1):3159 (2020)

APC/Cyanine7 anti-mouse CD25 Antibody recognizes CD25, 55 kD glycoprotein, expressed on activated T and B cells, thymocyte subset, pre-B cells, and T regulatory cells. This antibody is quality control tested by immunofluorescent staining with flow cytometric analysis. This antibody has been tested by flow cytometric analysis of mouse splenocytes. Alissafi T, et al. 2018. J Clin Invest. 128:3840

Biotin anti-mouse CD279 (PD-1) Antibody reacts with CD279, a 50-55 kD immunoglobulin superfamily member also known as programmed death-1 (PD-1), expressed on a subset of CD4-CD8- thymocytes and on activated T and B cells. This antibody is quality control tested by immunofluorescent staining with flow cytometric analysis. This antibody has been tested by flow cytometric analysis of mouse splenocytes. Martínez-López M et al. 2019. Immunity. 50(2):446-461

CD11c Antibody, anti-mouse, Clone N418, is specific for the mouse CD11c present in dendritic cells in lymphoid organs and blood, in Langerhans cells in the epidermis, in dendritic cell progenitors in the bone marrow, and in in vitro generated bone marrow–derived dendritic cells. In spleen and lymph node, CD11c is expressed at high levels on conventional CD11c+CD45R–mPDCA-1– dendritic cells, and at moderate levels on CD11c+CD45R+ mPDCA-1+ plasmacytoid dendritic cells. Clone N418 is reported to be weakly expressed on NK cells, B cells, and T cell subsets. It has been tested by flow cytometric analysis of mouse splenocytes. In order to compare the epitope specificity of an antibody, the clone being used is compared with other known clones recognizing the same antigen in a competition assay. Yang Z et al. USP12 downregulation orchestrates a protumourigenic microenvironment and enhances lung tumour resistance to PD-1 blockade. Nat Commun. 12(1):4852 (2021)

TCF1/TCF7 (C63D9) Rabbit mAb (Alexa Fluor® 488 Conjugate) detects endogenous levels of total TCF1/TCF7 protein. This antibody does not recognize the dominant negative isoforms of TCF1/TCF7 lacking the amino-terminal β-catenin binding domain and does not cross-react with LEF1. Willinger, T. et al. (2006) J Immunol 176, 1439-46.

CD11b Antibody, anti-mouse, Clone REA592, recognizes the mouse CD11b antigen (Mac-1 α; integrin αM chain), which is part of the CD11b/CD18 heterodimer (Mac-1 α, Mβ2 integrin), also known as the C3 complement receptor, expressed on monocytes, macrophages, and microglia. It has been tested by flow cytometric analysis of mouse splenocytes. Pahl, H. L. et al. (1992) Characterization of the myeloid-specific CD11b promoter. Blood (4) 79: 865 - 870

CD4 Antibody, anti-mouse, Clone REA604, recognizes the mouse CD4 antigen, a cell surface glycoprotein, which is a member of the immunoglobulin superfamily, expressed on T helper cells, regulatory T cells, and at lower levels on subpopulations of NKT cells and dendritic cells. In order to compare the epitope specificity of an antibody, the clone being used is compared with other known clones recognizing the same antigen in a competition assay. It has been tested by flow cytometric analysis of mouse splenocytes. Tourvieille, B. et al. (1986) Isolation and sequence of L3T4 complementary DNA clones: expression in T cells and brain. Science (4776) 234: 610 - 614

Granzyme B Antibody, anti-human/mouse/rat, Clone REA226, recognizes granzyme B, a 32 kDa member of serine protease family, which is involved in cell lysis mediated by cytotoxic T lymphocytes (CTLs) and NK cells. In order to compare the epitope specificity of an antibody, the clone being used is compared with other known clones recognizing the same antigen in a competition assay. It has been tested by flow cytometric analysis of mouse splenocytes. Grossman, W. J. et al. (2004) Differential expression of granzymes A and B in human cytotoxic lymphocyte subsets and T regulatory cells. Blood 104: 2840 - 2848

Ly-6C Antibody, anti-mouse, Clone REA796, recognizes the mouse Ly-6C antigen, which is a 14–17 kDa GPI-linked cell surface antigen that belongs to the Ly-6 family of murine surface glycoproteins. It is expressed on monocytes in the bone marrow and shortly after their migration into the circulation. In order to compare the epitope specificity of an antibody, the clone being used is compared with other known clones recognizing the same antigen in a competition assay. It has been tested by flow cytometric analysis of mouse splenocytes. Schlueter, A. J. et al. (1997) Distribution of Ly-6C on lymphocyte subsets: I. Influence of allotype on T lymphocyte expression. J Immunol 158: 4211 - 4222.

MHC Class II Antibody, anti-mouse, Clone REA813, ecognizes the MHC class II expressed on antigen-presenting cells, such as dendritic cells, monocytes/macrophages, B cells in lymphoid and non-lymphoid tissue. In order to compare the epitope specificity of an antibody, the clone being used is compared with other known clones recognizing the same antigen in a competition assay. It has been tested by flow cytometric analysis of mouse splenocytes. Bhattacharya, A. et al. (1981) A shared alloantigenic determinant on Ia antigens encoded by the I-A and I-E subregions: evidence for I region gene duplication. J Immunol (6) 127: 2488 - 2495

PE anti-mouse CD62L Antibody recognizes CD62L, a 74-95 kD glycoprotein also known as L-selectin, LECAM-1, Ly-22, LAM-1, and MEL-14, expressed on the majority of B and naïve T cells, a subset of memory T cells, monocytes, granulocytes, most thymocytes, and a subset of NK cells. This antibody is quality control tested by immunofluorescent staining with flow cytometric analysis. It has been tested by flow cytometric analysis of mouse splenocytes. Soon MSF, et al. 2020. Nat Immunol. 1.984027778.

Brilliant Violet 421™ anti-mouse CD152 Antibody reacts with CD152, also known as CTLA-4 or Ly-56, a 33 kD member of the immunoglobulin superfamily, expressed on activated T and B lymphocytes. This antibody is quality control tested by immunofluorescent staining with flow cytometric analysis. It has been tested by flow cytometric analysis of mouse splenocytes. Singh M, et al. 2017. Nat Commun. 8:1447.

Ly-6G Antibody, anti-mouse, REA526, recognizes the mouse lymphocyte antigen 6G (Ly-6G) antigen, a 21–25 kDa glycosylphosphatidylinositol (GPI)-linked protein which is highly expressed on neutrophilic granulocytes. In order to compare the epitope specificity of an antibody, the clone being used is compared with other known clones recognizing the same antigen in a competition assay. Tanaka, Y. et al. (2012) Stimulation of Ly-6G on neutrophils in LPS-primed mice induces platelet-activating factor (PAF)-mediated anaphylaxis-like shock. J. Leukoc. Biol. (3) 91: 485 - 494

CD45 Antibody, anti-mouse, REA737, recognizes the mouse CD45 antigen, also known as Ly-5 or leukocyte common antigen (LCA),involved in T cell receptor and B cell receptor signal transduction. It is expressed at high levels on all cells of hematopoietic origin except for erythrocytes. Clone REA737 reacts with all CD45 isoforms. In order to compare the epitope specificity of an antibody, the clone being used is compared with other known clones recognizing the same antigen in a competition assay. Arendt, C. W. et al. (1997) Identification of the CD45-associated 116 kDa and 80 kDa proteins as the alpha- and beta-subunits of alpha-glucosidase II. J. Biol. Chem. (20) 272: 13117 - 13125

F4/80 Antibody, anti-mouse, REA126, recognizes F4/80, a member of epidermal growth factor (EGF)-transmembrane 7 (TM7) family.

F4/80 is considered as one of the most specific cell-surface markers for murine macrophages. In order to compare the epitope specificity of an antibody, the clone being used is compared with other known clones recognizing the same antigen in a competition assay. Kortlever, R. M. et al. (2017) Myc Cooperates with Ras by Programming Inflammation and Immune Suppression. Cell (6) 171: 1301 - 1315

In vivo antibodies:
InVivoMAb anti-mouse OX40 (CD134) reacts with mouse OX-40 also known as CD134 and provides a costimulatory signal to an antigen-reacting naive T cells to prolong proliferation. In vivo treatment with an agonist antibody to OX-40 has been shown to strongly enhance the generation of antigen-specific effector T cells and prevent the induction of T cell tolerance. The OX-86 antibody is an agonistic antibody that has been shown to delay tumor growth in vivo. Krupnick, A. S., et al. (2014). "Central memory CD8+ T lymphocytes mediate lung allograft acceptance" J Clin Invest 124(3): 1130-1143.

InVivoMAb anti-mouse PD-L1 (B7-H1) reacts with mouse PD-L1 (programmed death ligand 1) also known as B7-H1 or CD274, expressed on T lymphocytes, B lymphocytes, NK cells, dendritic cells. In mouse models of melanoma, tumor growth can be transiently arrested via treatment with antibodies which block the interaction between PD-L1 and PD-1. The 10F.9G2™ antibody has been shown to block the interaction between PD-L1 and PD-1 and between PD-L1 and B7-1 (CD80). Stathopoulou, C., et al. (2018). "PD-1 Inhibitory Receptor Downregulates Asparaginyl Endopeptidase and Maintains Foxp3 Transcription Factor Stability in Induced Regulatory T Cells" Immunity 49(2): 247-263 e247.

InVivoMAb anti-mouse PD-1 (CD279) reacts with mouse PD-1 (programmed death-1) also known as CD279, expressed on CD4 and CD8 thymocytes as well as activated T and B lymphocytes and myeloid cells. In mouse models of melanoma, tumor growth can be transiently arrested via treatment with antibodies which block the interaction between PD-L1 and its receptor PD-1. The RMP1-14 antibody has been shown to block the binding of both mouse PD-L1-Ig and mouse PD-L2-Ig to PD-1.

InVivoPlus anti-mouse CTLA-4 (CD152) reacts with mouse CTLA-4 (cytotoxic T lymphocyte antigen-4) also known as CD152, expressed on activated T and B lymphocytes. CTLA-4 is among a group of inhibitory receptors being explored as cancer treatment targets through immune checkpoint blockade. The UC10-4F10-11 antibody has been shown to promote T cell co-stimulation by blocking CTLA-4 binding to the B7 co-receptors, allowing for CD28 binding. Honda, T., et al. (2014). "Tuning of antigen sensitivity by T cell receptor-dependent negative feedback controls T cell effector function in inflamed tissues" Immunity 40(2): 235-247.

IHC antibodies:

Anti-CD3 antibody [SP7] is suitable for staining normal and neoplastic T cells in formalin-fixed, paraffinembedded tissues. Cao B et al. Remodelling of tumour microenvironment by microwave ablation potentiates immunotherapy of AXL-specific CAR T cells against non-small cell lung cancer. Nat Commun 13:6203 (2022).

Anti-CD8 alpha antibody [EPR21769] ab217344 Identifies cytotoxic/suppressor T-cells that interact with MHC class I bearing targets and is Suitable for IHC-Fr, IHC-P. Wang L et al. DNA mechanical flexibility controls DNA potential to activate cGAS-mediated immune surveillance. Nat Commun 13:7107 (2022). Zhang P et al. Optimized dose selective HDAC inhibitor tucidinostat overcomes anti-PD-L1 antibody resistance in experimental solid tumors. BMC Med 20:435 (2022).

Recombinant Anti-Myeloperoxidase antibody [EPR20257] is is specific to Myeloperoxidase heavy chain and suitable for IHC-P and ICC/IF. Meng Y et al. Reactive metal boride nanoparticles trap lipopolysaccharide and peptidoglycan for bacteria-infected wound healing. Nat Commun 13:7353 (2022).

Biotin Rat Anti-Mouse CD19 reacts with CD19, a B lymphocyte-lineage differentiation antigen and is is suitable for IHC-P. Engel P, Zhou LJ, Ord DC, Sato S, Koller B, Tedder TF. Abnormal B lymphocyte development, activation, and differentiation in mice that lack or overexpress the CD19 signal transduction molecule. Immunity. 1995; 3(1):39-50.

Eukaryotic cell lines

Policy information about cell lines and Sex and Gender in Research

Cell line source(s)	B16-F10 and LLC1 cell lines were purchased from ATCC
Authentication	Cell lines were authenticated by STR profiling
Mycoplasma contamination	All cell lines were routinely tested for mycoplasma contamination and results were negative
Commonly misidentified lines (See ICLAC register)	None

Animals and other research organisms

Policy information about studies involving animals; ARRIVE guidelines recommended for reporting animal research, and Sex and Gender in Research

Laboratory animals	C57BL/6J female mice, 6–8 weeks old, were purchased from Charles River and housed under pathogen-free conditions at 22±2°C with 55±10% relative humidity and with 12h day/light cycles in Cogentech animal facility and with food and water ad libitum.
Wild animals	The study did not involve wild animals

Reporting on sex	In this study, we used only female mice in accordance with our animal protocols approved by the Institutional Animal Care and Use Committee (OPBA) at IFOM-The AIRC Institute of Molecular Oncology. However, these findings are independent of sex and valid for both females and males.
Field-collected samples	The study did not involve field-collected samples
Ethics oversight	All animal experiments were performed in accordance with the guidelines established in the Principles of Laboratory Animal Care (directive 86/609/EEC), were approved by the Italian Ministry of Health and were performed under the supervision of the institutional organism for animal welfare (Cogentech OPBA).

Note that full information on the approval of the study protocol must also be provided in the manuscript.

Flow Cytometry

Plots

Confirm that:

- [x] The axis labels state the marker and fluorochrome used (e.g. CD4-FITC).
- [x] The axis scales are clearly visible. Include numbers along axes only for bottom left plot of group (a 'group' is an analysis of identical markers).
- [x] All plots are contour plots with outliers or pseudocolor plots.
- [x] A numerical value for number of cells or percentage (with statistics) is provided.

Methodology

Sample preparation	For the flow cytometry analysis of tumor-infiltrating lymphocytes, tumors were minced, digested for 1 hour with Collagenase D<br>(10mg/ml) and DNAseI (10 mg/ml). Processed tumor were load on Lympholyte gradient and centrifuged at 1500 g for 30 minutes. The<br>interphase ring, which contains most live leukocytes cells, was collected and used for FACS staining. 1–2 × 106 cells per sample were<br>stained with the LIVE/DEAD stain (Invitrogen), and then with membrane protein marker (CD45, CD3, CD8, CD4, CD44) followed by fixation<br>with formaldehyde. For intranuclear (Foxp3, Tbet, Klrg1, Eomes, Tcf1/7) and cytoplasmic marker (GzmB) staining, cells were permeabilized<br>and fixed with Foxp3/transcription factor staining kit (Invitrogen eBioscence) or BD cytofix/cyto perm kit (BD bioscences).
Instrument	Data acquisition was performed on Attune NxT Flow Cytometer
Software	Results were analyzed with the FlowJo software
Cell population abundance	The entire cell population was analyzed without post sort analysis for each sample
Gating strategy	Gating strategy for CD8 lymphocyets and NK cells: Among viable CD45+ cells NK cells were gated as NKp46+ and T cells were gated as CD3+. Among T cells, CD8+ effector memory T cell were gated as CD44+ CD62L-.<br>Gating strategy for myeloid cells (Figure 3 A-D): Among CD45+ cells, dendritic cells (DC) were gated as CD11c+ MHCII+ and macrophages were gated as F4/80+ CD11b+. PMN-MDSC and M-MDSC cells were identified among F4/80 CD11b+ cells according to the expression of Ly6C and Ly6G.

- [x] Tick this box to confirm that a figure exemplifying the gating strategy is provided in the Supplementary Information.
